# Clinical literature on postoperative delirium and neurocognitive disorders: a historical systematic review

**DOI:** 10.1186/s44158-022-00039-6

**Published:** 2022-03-03

**Authors:** Gianluca Villa, Lorenzo Foti, Tessa Piazzini, Gaetano Russo, Marin Verrengia, Corinne Sangermano, Federico Bilotta, Stefano Romagnoli

**Affiliations:** 1 Department of Health Science Section of Anesthesiology and Intensive Care, University of Florence, Florence, Italy; 2 Department of Anesthesiology and Intensive Care, Azienda Ospedaliero-Universitaria Careggi, Florence, Italy; 3 Department of Anesthesiology and Intensive Care Medicine, Policlinico Umberto I, “Sapienza” University of Rome, Rome, Italy

**Keywords:** Postoperative delirium, Neurocognitive disorders, Surgery, Outcome

## Abstract

**Background:**

Since the appearance of the first report on postoperative cognitive impairment in 1955, the number of papers focusing on perioperative neurocognitive disorders (PND) has constantly increased, both in the field of basic science and clinical research. A critical comprehensive review may explore the perception of how noteworthy PND is for physicians and clinical researchers. The aim of this systematic review is to describe how the clinical papers published to date with PND as primary or secondary outcome have changed over time in terms of editorial characteristics.

**Results:**

A literature search was performed on PubMed, Embase, CINAHL, Cochrane, Scopus, and Web of Science databases, up to March 2021. Human prospective or retrospective clinical studies in which incidence, risk factors, treatments, or outcomes associated with PND are described among primary or secondary outcomes were included. A total of 2109 articles were considered.

**Conclusions:**

The bibliometric analysis suggests a stable increase in attention towards PND, particularly in general surgery adult-elderly patients, and underlines the importance for the clinicians not to underestimate this specific field.

**Supplementary Information:**

The online version contains supplementary material available at 10.1186/s44158-022-00039-6.

## Background

Perioperative neurocognitive disorders (PND) are frequent and severe complications occurring worldwide, particularly in elderly patients undergoing general anesthesia [1]. Indeed, postoperative delirium (POD) and postoperative cognitive dysfunction (POCD) are correlated with most of the memory impairments and executive and cognitive dysfunctions observed after surgery, both in the short and long term [1]. The associations between PND and worst surgical outcomes are nowadays clear, especially in case of unexpected intensive care unit (ICU) admission, prolonged ICU length of stay, patient hospitalization, and delayed surgical recovery [2–4]. PND is thus a major focus of current research in the field of perioperative medicine [5].

Since the first report on postoperative cognitive impairment in 1955 [6], interest in this spectrum of disorders has constantly increased, both in the field of basic science and clinical research. Over the last decades, pre-clinical experimental studies in animals and humans have tried to identify the neuro-psychiatric, pathophysiological mechanisms underlying PND. Moreover, several clinical studies worldwide have analyzed risk factors, diagnostic tools, and treatment strategies for PND in a broad variety of surgical settings [1]. Nonetheless, POD and POCD are still generally underestimated, underdiagnosed, and undertreated in current bedside practice [7].

### Purpose of review

The evaluation of the perception of PND in the different clinical settings is certainly an issue that deserves more research attention. A critical comprehensive literature review, pruned by basic-science and translational medicine, may explore the perception of how noteworthy PND is for physicians and clinical researchers. The aim of this systematic review is to describe how the clinical papers published to date with PND as primary or secondary outcome have changed over time in terms of editorial characteristics.

## Methods

This systematic review has been recorded in the International Prospective Register Of Systematic Review (PROSPERO registration number: CRD42021246906).

A literature search on PubMed, Embase, CINAHL, Cochrane, Scopus, and Web of Science databases was conducted up to March 2021, using the following keywords: “delirium”, “confusion”, “disorientation”, “bewilderment”, “postoperative”, “surgery”, and “anesthesia recovery” (see Additional file 1).

Removal of duplicates was performed automatically, through a bibliographic management software (Mendeley), and then reviewed manually. Two researchers (GR and LF) independently screened titles and abstracts of the publications to identify papers in which incidence, risk factors, treatments, or outcomes associated with PND were described among primary or secondary outcomes. Papers describing preclinical studies (e.g., molecular or histologic studies, experimental animal models, or drugs discovery) were excluded from eligibility. All (human) prospective or retrospective clinical studies about PND, with POD and POCD as the main clinical manifestations, were analyzed according to descriptive characteristics, such as language, age of population, type of publication, and surgical setting. All these variables were reported in an electronic reporting system and organized systematically. Finally, the reference section of retrieved systematic reviews and meta-analyses was analyzed in order to identify potentially eligible articles that had not been identified through the literature search.

We analyzed the number of articles with PND as the main topic published annually over time, as well as variation over time of specific descriptive characteristics, i.e., language and type of publication, age of the enrolled population, and surgical setting. The language of publication has been described as English vs other languages, while the age of the enrolled population as pediatric or mixed adult/pediatric vs adult surgical patients. The types of publications have been divided into four groups and described as (1) observational (prospective or retrospective) studies and randomized clinical trials (RCTs), (2) systematic reviews and meta-analyses, (3) case reports and editorials, and (4) preliminary protocols of RCTs or observational studies exploring PND as the main outcome. The types of surgery have been divided into four groups and described as (1) general surgery (including articles describing major abdominal, thoracic, orthopedic, vascular, spine, and endocrinologic procedures), (2) brain surgery (including articles describing brain surgery alone and those considering brain surgery besides other types of surgery), (3) cardiac surgery (including articles describing cardiac surgery alone and those considering cardiac surgery besides other types of surgery), and (4) another group including articles considering mixed types of surgical procedures (e.g., general and cardiac and brain surgery).

## Results

**Fig. 1:**
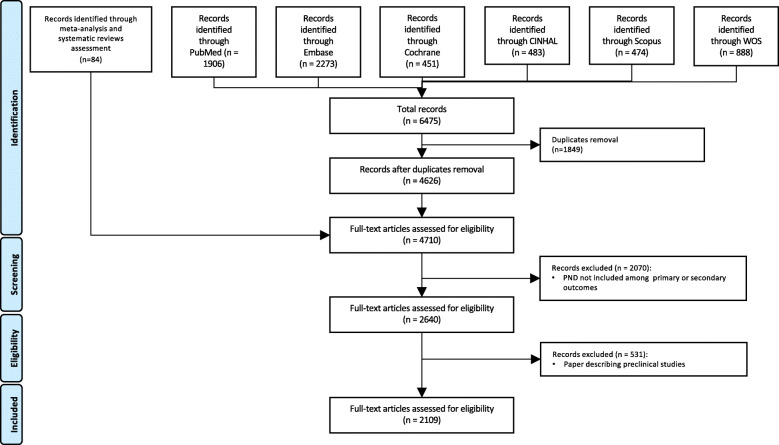
Flow chart describing the studies selection process

**Fig. 2:**
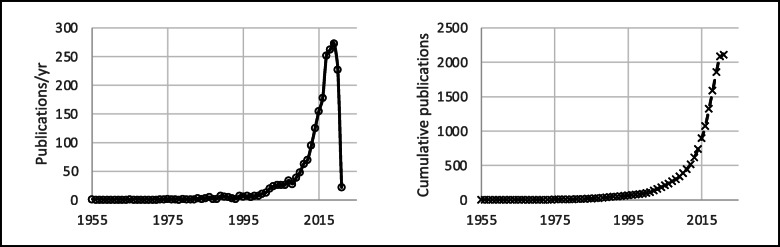
Number of papers on PND published annually (left) and cumulatively (right) from 1955 to date

**Fig. 3:**
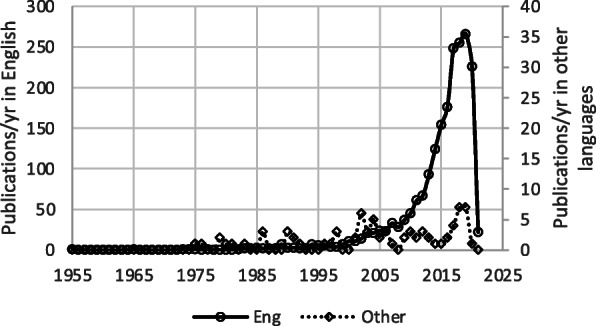
Number of papers on PND published annually from 1955 to date in English and other languages

**Fig. 4:**
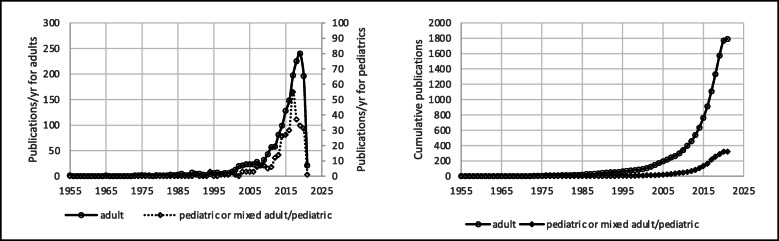
Number of papers on PND published annually (left) and cumulatively (right) including adult or pediatric-mixed adult/pediatric surgical patients

**Fig. 5:**
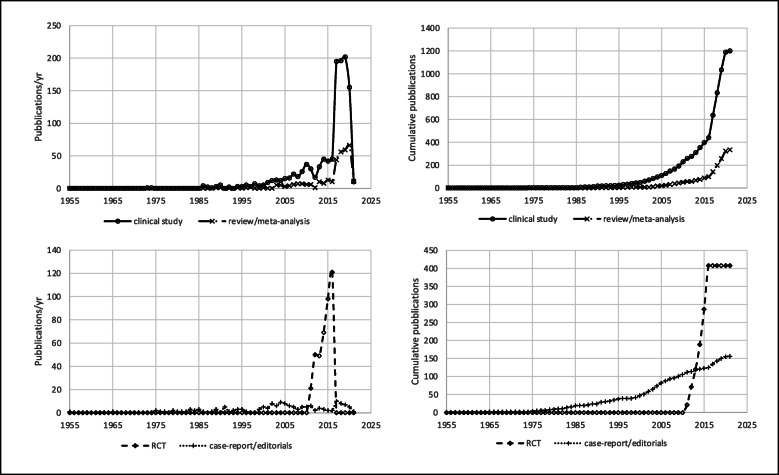
Number of papers on PND published annually (left) and cumulatively (right) according to type of publication

**Fig. 6:**
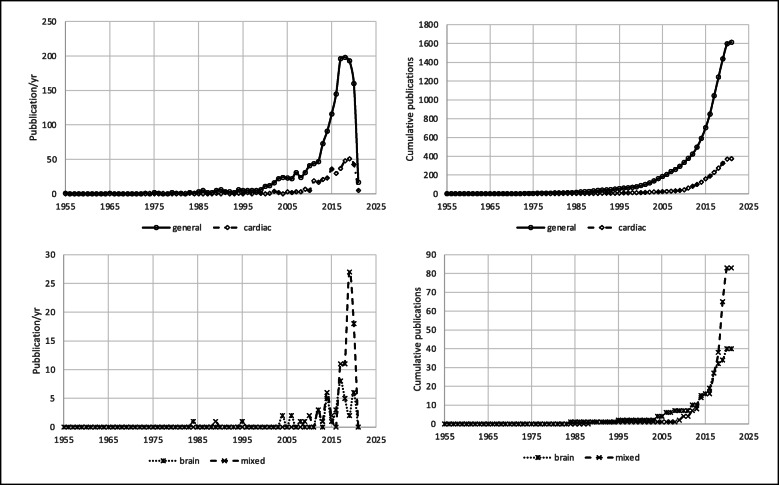
Number of papers on PND published annually (left) and cumulatively (right) according to surgical setting

The literature search was performed between April and May 2021. The database search identified 6475 articles. After removal of duplicates (*n* = 1849), 4626 articles remained. The analysis of reference sections of retrieved systematic reviews and meta-analysis resulted in additional 82 studies for a total of 4710 articles. Of these, 2601 were excluded because PND was not included among the primary or secondary outcomes. A total of 2109 articles were thus considered for this systematic review (Fig. 1). Figure 2 displays annual and cumulative numbers of publications published to date. Cumulatively, 2031 articles on PND have been published in English since 1955. Variation over time in the language of publication is described in Fig. 3. Cumulatively, 1789 papers have described PND in adult surgical populations, while the remaining 320 have considered pediatric or mixed adult/pediatric surgical patients. The first article focusing on a pediatric population appeared in the literature in 1990. Variation over time in the age of the enrolled population is described in Fig. 4, in terms of annual and cumulative numbers of publications. PND was examined in 1199 articles, including both observational studies (prospective or retrospective) and RCTs. PND was the main topic of discussion of 334 meta-analyses or systematic reviews and 156 case reports or editorials. Four hundred eight articles were preliminary protocols of RCTs or observational studies exploring PND as the main outcome. Figure 5 describes the annual and cumulative numbers of publications according to type of publication. The main types of surgical procedures described in the selected publications were abdominal, thoracic, orthopedic, vascular, spine, and endocrinologic surgeries (1613 articles). Cardiac surgery (alone or in combination) and brain surgery were described in 373 and 40 articles, respectively. Eighty-three articles could not be classified because they included a mix of different general, brain, and cardiac surgery procedures. Figure 6 describes the annual and cumulative numbers of publications according to surgical settings.

## Discussion

In this systematic review, we analyzed the clinical papers on PND published up to March 2021. Since the publication of the first report in 1955, there has been increasing attention to this specific field, particularly in general surgery adult-elderly patients.

Results available from observational and comparative studies along with published protocols of ongoing RCTs on surgical patients reveal that PND is an issue of major concern to the clinical academic world. Nevertheless, the growing scientific interest on PND seems to diverge from the disheartening observational data on real-life practice and physicians’ attitude to routinely test patients for POD or POCD [8]. As an example, an on-line survey endorsed by the European Society of Anesthesiology has demonstrated that, despite PND (and particularly POD) being perceived as a relevant condition, only 7% of physicians involved in perioperative care who responded to the survey routinely monitor the majority of their patients for delirium [7]. Apparently, academic interest rather than clinical practice has encouraged the production of scientific publications on PND, as demonstrated by their stable increase over time.

This review underlines the importance for the clinicians to pay attention to this really frequent disorder that can affect surgical patients.

A stable increase in papers on PND research in the last decades has already been observed by Mi et al. in a bibliometric analysis aimed at identifying the top 100 cited publications on PND. In this paper, the authors considered both experimental and basic science papers along with clinical (observational and comparative) studies. Within this context, the steadily growing number of studies on PND reported by the authors may reflect the interest of physicians/clinical researchers, as well as of basic science scientists. In order to focus our attention on the clinical perception of PND, pared down from basic science and pre-clinical interests, we restricted our analysis to studies that may originate from bedside practice. Interestingly, our study reveals the same increasing trend in numbers of publications on PND over time. Indeed, our systematic review describes an unexpected evolution of the interest toward a concrete clinical conception of PND that involves not only academic attention but also bedside clinical practice. This aspect has probably determined the peculiar profile of the publication curve displayed in Fig. 2. In particular, the number of papers published in the literature seems to be influenced by specific clinical research milestones in the field of PND, such as the ISPOCD1 study (published in *Lancet* in 1998 [9]) or the longitudinal assessment of neurocognitive function after cardiac surgery (published in the *New England Journal of Medicine* in 2001 [10]), or the paper published in 2018 examining the nomenclature on perioperative cognitive impairment and leading to the inclusion of PND among definitive neurocognitive disorders listed in the DSM-5 manual [5]. The same trend can be observed in the subgroup of papers considering pediatric populations (Fig. 4), mainly polarized around the GAS trial published in *Lancet* in 2016 [11]. Interestingly, Mi et al. have recognized these papers as the most cited articles focusing on cognitive changes associated with anesthesia and/or surgery [1].

The high prevalence of papers analyzed by Mi et al. and published in journals with an anesthesiological or surgical attraction is probably related to the search strategy adopted by the authors, confined to Web of Science database. In our bibliometric study, to better characterize the interest that a whole multidisciplinary and multiprofessional team may have in managing patients with PND, we have adopted a different search strategy encompassing several different databases, including also PubMed, Embase, CINAHL, or Cochrane [12]. This field appears to be of particular interest to nurses, biomedical engineers, and clinical pharmacologists who have published several clinical papers in the last decades on the potential effects on PND of new drugs, disposables, or technologies nowadays routinely available at the bedside. The interest of industries and regulatory agencies has followed accordingly. The number of papers with funding disclosures has thus increased over time [1]. All these aspects may have further contributed to the transition from basic science and preclinical interest to a more pragmatic, clinical, bedside, patients-centered research on PND.

Compared to previous bibliometric studies available for postoperative cognitive impairment, the adoption of multiple databases [13] and a refined search strategy have contributed to guarantee an adequate and efficient coverage of the topic in this systematic review. Our search strategy was in line with that used by the most updated guidelines on POD published in 2017 by the European Society of Anesthesiology.

Several drawbacks can be recognized in this study. First, although several articles included in this systematic review designate POD or POCD as their main focuses, the authors miss to define the clinical tools adopted to diagnose these conditions. Most papers indeed fail to use any validated scales to diagnose POD or POCD, potentially leading to inaccuracies and biases in the final bibliometric analysis. Second, the high prevalence of papers describing PND in general surgery patients can be influenced by the inclusion of orthopedic surgery in the “general surgery” group. Finally, due to the considerable number of articles screened and included in this systematic review and the impossibility to automatically determine the number of citations or the journal title during data extraction, we could not report this important bibliometric information in our analysis.

## Supplementary Information

**Additional file 1.** Literature search.

## Data Availability

Literature search strings are listed in Additional file 1.

## Abbreviations

PND: Perioperative neurocognitive disorders
POD: Postoperative delirium
POCD: Postoperative cognitive dysfunction
ICU: Intensive care unit
RCTs: Randomized clinical trials
