# Supplementary material for: Clinical literature on postoperative delirium and neurocognitive disorders: a historical systematic review
Source: J Anesth Analg Crit Care. 2022 Mar 3;2:11. doi: 10.1186/s44158-022-00039-6 (PMC10246356; doi:10.1186/s44158-022-00039-6)
Supplement: Supplementary file 1 — Additional file 1. Literature search. [file 44158_2022_39_MOESM1_ESM.pdf]

1.- PubMed:

(Delirium OR delirious OR confusion OR disorientation OR bewilderment) AND (Postoperative OR "post operative" OR postintervention OR "post intervention" OR "post surgical" OR postsurgical OR "post surgery" OR postsurgery OR "anesthesia recovery" OR "anaesthesia recovery" OR "Anesthesia Recovery Period"[Mesh] OR postanesthesia OR "post anesthesia" OR "post anaesthesia")

2.- EMBASE:

((('delirium'/exp OR delirium OR delirious OR 'confusion'/exp OR confusion OR 'disorientation'/exp OR disorientation OR bewilderment) AND ('postoperative complication'/exp OR postoperative OR 'post operative' OR 'postoperative period'/exp OR postintervention OR 'post intervention' OR 'post surgical' OR postsurgical OR 'post surgery' OR postsurgery OR 'anesthesia recovery'/exp OR 'anesthesia recovery' OR 'anaesthesia recovery'/exp OR 'anaesthesia recovery' OR postanesthesia OR 'post anesthesia' OR 'post anaesthesia')) OR 'postoperative delirium'/exp) AND ([cochrane review]/lim OR [systematic review]/lim OR [meta analysis]/lim OR [randomized controlled trial]/lim OR 'observational study' OR 'case study')

3.- CINAHL:

(Delirium OR delirious OR confusion OR disorientation OR bewilderment) AND (Postoperative OR "post operative" OR postintervention OR "post intervention" OR "post surgical" OR postsurgical OR "post surgery" OR postsurgery OR "anesthesia recovery" OR "anaesthesia recovery" OR postanesthesia OR "post anesthesia" OR "post anaesthesia")

Limiters - Publication Type: Case Study, Meta Analysis, Randomized Controlled Trial, Systematic Review

OR

((Delirium OR delirious OR confusion OR disorientation OR bewilderment) AND (Postoperative OR "post operative" OR postintervention OR "post intervention" OR "post surgical" OR postsurgical OR "post surgery" OR postsurgery OR "anesthesia recovery" OR "anaesthesia recovery" OR postanesthesia OR "post anesthesia" OR "post anaesthesia")) AND (observational study OR observational research)

OR

(MH "Delirium" OR MH "Confusion+") AND ((MH "Postoperative Complications") OR (MH "Postoperative Period"))

Limiters - Publication Type: Case Study, Meta Analysis, Randomized Controlled Trial, Systematic Review

OR

(MH "Delirium" OR MH "Confusion+") AND ((MH "Postoperative Complications") OR (MH "Postoperative Period")) AND ((observational study OR observational research))

4.- COCHRANE:

#1 ((Delirium OR delirious OR confusion OR disorientation OR bewilderment) AND (Postoperative OR "post operative" OR postintervention OR "post intervention" OR "post surgical" OR postsurgical OR "post surgery" OR postsurgery OR "anesthesia recovery" OR "anaesthesia recovery" OR postanesthesia OR "post anesthesia" OR "post anaesthesia")):ti,ab,kw 1829

#2 MeSH descriptor: [Delirium] explode all trees 765

#3 MeSH descriptor: [Confusion] explode all trees 899

#4 #2 OR #3 899

#5 MeSH descriptor: [Postoperative Period] explode all trees 5872

#6 MeSH descriptor: [Postoperative Complications] explode all trees 39635

#7 MeSH descriptor: [Anesthesia Recovery Period] explode all trees 2031

#8 #5 OR #6 OR #7 43861

#9 #4 AND #8 326

#10 #1 OR #9 1853

5.- SCOPUS:

TITLE-ABS (delirium OR delirious OR confusion OR disorientation OR bewilderment) AND TITLE-ABS (postoperative OR {post operative} OR postintervention OR {post intervention} OR {post surgical} OR postsurgical OR {post surgery} OR postsurgery OR {anesthesia recovery} OR {anaesthesia recovery} OR postanesthesia OR {post anesthesia} OR {post anaesthesia}) AND TITLE-ABS ({systematic review} OR {case series} OR {randomized controlled trial} OR rct OR {meta analysis} OR metanalysis OR {observational study})

6.- WEB OF SCIENCE:

TITLE-ABS (delirium OR delirious OR confusion OR disorientation OR bewilderment) AND TITLE-ABS (postoperative OR {post operative} OR postintervention OR {post intervention} OR {post surgical} OR postsurgical OR {post surgery} OR postsurgery OR {anesthesia recovery} OR {anaesthesia recovery} OR postanesthesia OR {post anesthesia} OR {post anaesthesia}) AND TITLE-ABS ({systematic review} OR {case series} OR {randomized controlled trial} OR rct OR {meta analysis} OR metanalysis OR {observational study})
